# Development and validation of a work-related risk score for upper-extremity musculoskeletal disorders in a French working population

**DOI:** 10.5271/sjweh.4119

**Published:** 2023-11-01

**Authors:** Clémence Rapicault, Yves Roquelaure, Julie Bodin, Natacha Fouquet, Sandrine Bertrais

**Affiliations:** 1 Univ Angers, CHU Angers, Univ Rennes, Inserm, EHESP, Irset (Institut de recherche en santé, environnement et travail) - UMR_S 1085, SFR ICAT, Angers, France.; 2 Santé publique France, French national public health agency, F-94410 Saint-Maurice, France.

**Keywords:** France, musculoskeletal disease, occupational exposure, prediction model, risk assessment, work

## Abstract

**Objectives:**

The aim of this study was to develop an easy-to-use risk score based on occupational factors and to validate its performance to identify workers either having (diagnostic setting) or developing (prognostic setting) upper-extremity musculoskeletal disorders (UEMSD).

**Methods:**

This study relied on data from the Cosali prospective cohort conducted in a French working population. Diagnostic status for six UEMSD at inclusion and at follow-up was assessed by a standardized clinical examination. Occupational factors were collected through a self-administered questionnaire completed before the clinical examination at inclusion. The risk score was derived from a multivariate penalized logistic regression model on data of 2468 workers included in 2002–2003 (323 UEMSD cases), and the diagnostic validation of the score was performed using data of 1051 workers included in 2004–2005 (126 UEMSD cases). The score’s performance for predicting UEMSD at follow-up in workers without UEMSD at baseline was then assessed.

**Results:**

The risk score includes physical, psychosocial and organizational factors at work. In the diagnostic validation sample, it had acceptable calibration and discrimination performance for UEMSD at baseline, with a relatively low area under the ROC curve (AUC) (0.60, 95% confidence interval 0.57–0.63) and a high negative predictive value (89–90%). The baseline risk score showed similar performance in prognostic setting.

**Conclusion:**

This UEMSD risk score was developed with the purpose to stratify work situations and prioritize those requiring intervention by an ergonomist. Further external validation studies are required to confirm its predictive performance and determine its practical utility as a first-line risk assessment tool, especially among working populations with higher prevalence of UEMSD.

Musculoskeletal disorders (MSD) are one of the most common work-related health problems in Europe and the leading cause of recognized occupational diseases (1). MSD are painful diseases that affect a large number of workers in almost all sectors of activity. They have costly consequences at individual and societal levels (limitation in activities, health care costs, loss of income, work disability, etc), and for companies (lower productivity, higher absenteeism, etc).

Upper-extremity MSD (UEMSD) represented >80% of all occupational diseases recognized in 2019 in France, with >40 500 cases (2). All musculoskeletal disorders, including UEMSD, are multifactorial diseases. It is recognized that the prevalence is higher among women, and that it increases with age regardless of gender (3). However, a large proportion of the UEMSD is attributable to occupational exposures, in particular to physical and psychosocial factors (4). Most workers are exposed to a combination of these factors, which increases their risk even more (4). As a result, it was estimated that a significant number of incident cases could potentially be avoided by preventing and reducing exposures to these work-related factors (4, 5).

MSD prevention is an occupational health priority in most countries. The primary prevention strategy is based on risk assessment – the purpose being to identify workers at risk due to their occupational exposures – and exposure control. Many tools are available to assess the risk of MSD in the workplace (6) and some of them provide a quantitative risk score based on physiological or ergonomic criteria (eg, OCRA checklist) (7). However, these risk assessment tools and approaches are mostly time consuming (eg, observations by ergonomists and work health and safety professionals), and require training and expertise (8). In addition, most methods focus only on physical risks while other work-related factors may contribute to the risk of MSD – and UEMSD in particular – namely psychosocial and organizational factors. Thus, it seemed necessary to provide work health and safety professionals a simple and easy-to-use tool for identifying the work situations that could benefit as a priority from preventive intervention action.

The main objective of this study was therefore to develop and validate a new practical scoring system quantifying occupational exposure to the risk of UEMSD, taking into account physical, psychosocial and organizational factors. The second aim was to evaluate the ability of this score to predict incident UEMSD.

## Methods

### Study design and population

The present study relied on the existing data of the COSALI prospective cohort, which was conducted from 2002 to 2010 in the general working population of the Pays de la Loire region in western France, as part of the National Musculoskeletal Disorders Surveillance Program among the working population (9, 10). Details of this study have been reported elsewhere (9–12). Briefly, 83 occupational physicians (OP) volunteered to randomly enroll workers when they came for a medical check-up, which was mandatory at least every two years for all workers according to the French Labor Code during the study period. Subjects were randomly selected using a two-stage sampling procedure. First, the investigators chose 15–30 half-days for each OP. Then, each OP was asked to randomly select 1 out of 10 workers from the scheduled visits on the selected half-days (10). The inclusion criteria for the COSALI study were as follows: workers aged 20–59 years whose medical surveillance was ensured by an OP participating in the network, working in a private or public company located in the Pays de la Loire region (even if the head office of the company is located outside this region), whatever the type of employment contract (permanent contract, fixed-term contract), suffering or not from MSD, benefiting or not from recognition of an occupational illness (10). More than 90% of the workers invited to participate gave their consent for the COSALI study. A total of 2513 workers were included during the first inclusion period (2002–2003), and 1197 additional workers were included in the second (2004–2005). A follow-up medical examination was conducted among 1611 participants between 2007 and 2010. The COSALI study received approval from the French ethics committees (CCTIRS N°01-215 and CNIL N°901 273).

### Outcome

The presence of UEMSD was assessed at inclusion in the cohort through a standardized OP-conducted clinical examination. The diagnostic process was based on the SALTSA European consensus-based criteria and clinical tests to evaluate work-related UEMSD (13). All OP previously received guidelines and a 3-hour training. Cases of UEMSD were defined as workers having one of the six following diagnosed UEMSD: (i) rotator cuff syndrome, (ii) lateral epicondylitis, (iii) carpal tunnel syndrome, (iv) ulnar cubital syndrome, (v) flexor-extensor peritendinitis or tenosynovitis of the forearm-wrist region, or (vi) De Quervain’s disease. The clinical examination at follow-up used the same standardized clinical diagnostic procedures to evaluate the presence of UEMSD.

### Candidate predictors

In addition to age and gender, a set of 47 occupational risk factors were selected as potential predictors of UEMSD (supplementary material, www.sjweh.fi/article/4119, table S1) using background knowledge, literature reviews (3) and previous results on the Cosali Cohort (5, 11, 12, 14, 15). Individual data on these occupational factors were collected through a self-administered questionnaire at baseline, completed by workers before their clinical OP examination.

Exposures to physical factors were defined using the SALTSA European consensus criteria (13): repetitiveness of tasks (≥4h/day) with/without break, arms above shoulder level (≥2h/day), wrist twisting movements (≥2h/day), arms abduction (60–90°) (≥2h/day), holding hand behind the trunk (≥2h/day), elbow flexion/extension movements (≥2h/day), pronation and supination movements (≥2h/day), use of the pinch grip (≥4h/day), use of vibrating hand tools (≥2h/day), use of computer keyboard or mouse (≥4h/day), exposure to cold temperature i.e. less than 15°C (≥4 h/day). Perceived physical exertion was assessed using the rating perceived exertion (RPE) Borg scale, which ranges from 6 (no exertion at all) to 20 (maximal exertion) (16). Workers were classified into 3 categories (RPE<13, 13 ≤RPE ≤15, RPE >15) according to thresholds proposed by the French National Research and Safety Institute for the Prevention of Occupational Accidents and Diseases (17).

Psychosocial factors at work were assessed using the validated French version of the Karasek’s Job Content Questionnaire (18). The scores and then the items of the three main dimensions were tested as candidate predictive variables: psychological demands (9 items), decision latitude (9 items), and social support (8 items). Items were tested into two groups defined as follows: strongly disagree/disagree versus strongly agree/agree.

Work organizational factors included five binary variables describing potential determinants of the participant’s work pace (dependent on automatic rate, dependent on production standards or deadlines, imposed by permanent monitoring, dependent on colleague’s work, dependent on external demand). Questions from national surveys on working conditions were used to collect these data (19). Two factors characterizing work schedule (shift work, irregular working hours), and two factors related to employment status (temporary employment, work with temporary workers) were also retained as candidates.

### Statistical analysis

**Figure 1:**
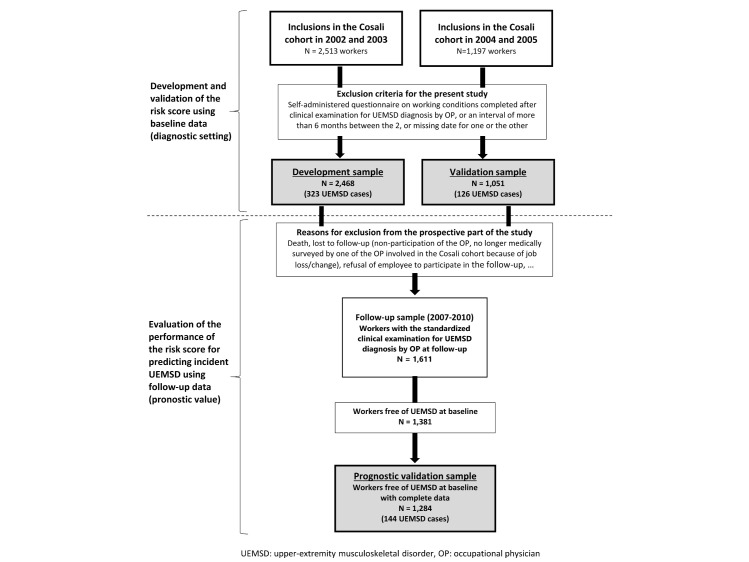
Flow-diagram for the study samples. [UEMSD=upper-extremity musculoskeletal disorder; OP=occupational physician.]

*Sample size.* All participants of the Cosali cohort who completed the self-administered questionnaire on working conditions during the six months preceding the clinical examination for UEMSD diagnosis were included. The sample used for the development of the prediction model (development sample) consisted of 2468 workers (including 323 workers with UEMSD) enrolled during the first inclusion period ie, 2002–2003, and the validation sample comprised 1051 workers (including 126 workers with UEMSD) enrolled during the second inclusion period ie, 2004–2005 (figure 1).

*Missing data.* Missing values for candidate variables were handled with multiple imputation by chained equations using the R package *mice*, since preliminary analyses suggested that a missing at random (MAR) hypothesis could be assumed. The same imputation process was applied separately in the development and validation samples. The outcome variable was included in the imputation process of candidate predictors for both samples (20), but we did not impute missing outcome values. As recommended by White & Royston (21), 20 imputed sets were generated for both development and validation samples since there were approximately 20% incomplete cases considering the 47 candidate predictor variables.

*Development of the work-related UEMSD risk score.* As indicated in figure 1, the UEMSD risk score was developed using baseline data (occupational exposures, OP-diagnosed UEMSD) of the workers recruited during the first wave of inclusion in 2002–2003. A three-step procedure was conducted for building the risk score. Least absolute shrinkage and selection operator (Lasso) was used to perform both predictor selection and overfitting correction through shrinkage of coefficients, ie, this penalized regression method reduces coefficients toward zero, so only the most important predictors remain in the model and their coefficients are corrected for optimism bias.

A first Lasso logistic regression was performed separately on each of the 20 imputed data sets for selecting predictors of UEMSD among the candidate variables. In each data set, the optimal value of lambda was automatically determined through cross-validation. Group Lasso approach was used to deal with variables with more than two categories, ie, to include all dummy variables in the model when at least one was selected. Age and gender were forced into the models as personal characteristics, thus the 47 occupational factors were tested as predictors of UEMSD taking into account potential differences in disease prevalence explained by age and gender. The final set of selected predictors was chosen based on the frequency of inclusion of the variables in the models obtained in the 20 sets of imputed data (22). Retained variables were those included in ≥15 of the 20 models (75%).

Then a Lasso logistic regression including only the selected predictor variables was applied to each imputed data set for estimating shrunk coefficients. Rubin’s rules (23) were used to combine the 20 estimates of the shrunk coefficients of the predictors, giving the final coefficients of the selected predictors in the model.

Finally, to create a risk score that can be easily and quickly calculated, each coefficient of the final model was multiplied by 10 and rounded to the nearest integer.

*Performance measures.* The overall performance of the score was measured with the Nagelkerke’s R^2^ and the scaled Brier score. The Nagelkerke’s R^2^ quantifies the amount of variance in the outcome accounted by the score. The scaled Brier score is an alternative to the R^2^ which measures accuracy of predictions, varying from 0% (perfect model) to 100% (worst model).

The calibration of the model was evaluated using a calibration plot of observed outcome versus predicted probabilities, and estimation of the calibration slope and intercept. A correct calibration corresponds to values around 1 for the slope and 0 for the intercept. The Hosmer-Lemeshow goodness-of-fit test was additionally performed.

The discriminative ability of the score was assessed using the area under the ROC curve (AUC), and measures of classification ability were calculated (diagnostic accuracy, sensitivity, specificity, positive and negative predictive values) at different score cut-offs. The discrimination slope, defined as the absolute difference in mean of predictions between subjects with and without the outcome was also used to evaluate the efficiency of the score to separate the two groups.

All these performance measures were estimated on each imputed data set and the results were combined using Rubin’s rules (24) and *micombine.chisquare* function for Hosmer-Lemeshow tests from *miceadds* R package.

*Validation of the developed UEMSD risk score.* The validation of the score was first performed in a diagnostic setting as in it was developed. The predictive score was applied to the baseline data of workers recruited in 2004–2005 to determine calibration, discrimination and measures of classification performance for UEMSD diagnosis at inclusion in this independent sample.

To explore deviation from the MAR hypothesis, a sensitivity analysis was conducted by assessing the score performance in the 968 workers of the validation sample with complete data for variables included in the prediction model.

We further performed a prognostic validation of the UEMSD risk score by calculating the risk score at inclusion and assessing its performance for predicting incidents cases of UEMSD clinically diagnosed at follow-up examination. The prognostic validation sample included 1381 workers, free of UEMSD at baseline, and with the standardized clinical examination for UEMSD diagnosis by OP at follow-up. And among them, 1284 had complete data.

*Additional sensitivity analyses.* The prognostic ability of the developed score was assessed separately for the three main locations of UEMSD, which are shoulder, elbow and hand/wrist. For these analyses, shoulder MSD corresponds to clinically diagnosed rotator cuff syndrome. Elbow MSD include clinically diagnosed lateral epicondylitis and cubital tunnel syndrome, and hand/wrist MSD take into account clinically diagnosed carpal tunnel syndrome, finger flexor tendinitis and de Quervain’s tenosynovitis.

All the statistical analyses were performed using R version 4.1.2 (R Project for Statistical Computing).

## Results

The general characteristics of development and validation samples are presented and compared in table 1. There were no significant differences between the two samples, including for the prevalence of UEMSD: 13.1% workers in the development sample and 12.0% workers in the validation sample had UEMSD at inclusion in the cohort. The comparison of occupational exposures between the samples is provided in supplementary table S1.

**Table 1:** Characteristics of the development and validation samples at inclusion in the Cosali cohort. [SD=standard deviation; UEMSD=upper-extremity musculoskeletal disorder]

		Development sample (N=2468)			Validation sample (N=1051)		P-value ^a^
		Mean (SD)	N (%)		Mean (SD)	N (%)	
Clinically diagnosed UEMSD			323 (13.1)			126 (12.0)	0.371
Age (years)		38.7 (10.4)			38.6 (10.2)		0.765
Gender (male)			1442 (58.4)			605 (57.6)	0.635
Occupational category							
	Missing		1			3	
	Professionals/managers		203 (8.2)			86 (8.2)	0.849
	Associate professionals/technicians		536 (21.7)			241 (23)	
	Clerks/service workers		652 (26.4)			277 (26.4)	
	Blue-collar workers		1076 (43.6)			444 (42.4)	
Economic sector							
	Missing		0			3	
	Agriculture		48 (1.9)			22 (2.1)	0.421
	Industries		806 (32.7)			358 (34.1)	
	Construction		150 (6.1)			50 (4.8)	
	Trade and services		1464 (59.3)			618 (59.0)	

^a^ Student’s t-test for age, Chi-Square test for categorical variables.

### Model development and validation

Table 2 shows the 13 candidate variables (6 physical exposures, 3 psychosocial work variables and 4 factors related to work organization) retained in the final predictive model for UEMSD, and their regression coefficients. The corresponding scoring system theoretically varies between 0 and 35.

**Table 2:** Final predictive model and scoring system derived from penalized logistic regression (Lasso) models on the 20 imputed datasets. [RPE=rating perceived exertion]

			Regression coefficients ^a^	Scoring system ^b^
**Physical factors**				
	Task repetitiveness (≥4h/day)	No	- ^c^	0
		Without break	0.31	3
		With break	0.50	5
	Perceived physical exertion	No	-	0
		13 ≤RPE Borg scale ≤15	0.37	4
		RPE Borg scale >15	0.48	5
	Arms above shoulder level	No or <2h/day	-	0
		≥2h/day	0.19	2
	Arms abduction (60–90°)	No or <2h/day	-	0
		≥2h/day	0.16	2
	Elbow flexion / extension movements	No or <2h/day	-	0
		≥2h/day	0.40	4
	Wrist twisting movements	No or <2h/day	-	0
		≥2h/day	0.26	3
**Psychosocial work factors ^d^**				
	Psychological demands: not enough time	No	-	0
		Yes	0.13	1
	Social support: superior not concerned	No	-	0
		Yes	0.31	3
	Social support: unhelpful co-workers	No	-	0
		Yes	0.38	4
**Organizational factors**				
	Irregular working hours	No	-	0
		Yes	0.13	1
	Work with temporary workers	No	-	0
		Yes	0.20	2
	Work pace dependent on colleague’s work	No	-	0
		Yes	0.18	2
	Work pace dependent on production standards or deadlines	No	-	0
		Yes	0.08	1
**Maximum score**				35

^a^ Adjusted for age and gender. Rubin’s rules were used to combine the shrunk coefficient estimates of each predictor obtained in the 20 imputed datasets. ^b^ Each regression coefficient was multiplied by 10 and rounded to the nearest integer. ^c^ Reference category. ^d^ Items from the Karasek Job Content Questionnaire (18).

Estimates of the performance of the developed risk score for identifying workers with UEMSD diagnosed at inclusion into the cohort (diagnostic performance) are given in table 3. Discrimination of the score was AUC 0.68, 95% confidence interval (CI) 0.66–0.69 in the development sample and AUC 0.60, 95% CI 0.57–0.63 in the validation sample. The score showed good calibration as reflected by calibration intercept and calibration slope. The non-significant Hosmer-Lemeshow test also indicated an adequate fit between the predicted probabilities and observed outcome data.

**Table 3:** Performance of the risk score for identifying workers with UEMSD diagnosed at inclusion into the cohort. Values were obtained by applying Rubin’s rules to combine performance measure estimates obtained in the 20 imputed datasets and micombine.chisquare function from miceadds R package for Hosmer-Lemeshow tests (24). [AUC=area under the ROC curve; CI=confidence interval.]

		Development sample (N=2468)	Validation sample (N=1051)
Overall performance			
	Nagelkerke’s R^2^	8.4%	2.5%
	Brier score	0.11	0.10
	Scaled Brier score	5.0%	1.4%
Discrimination			
	AUC (95% CI)	0.68 (0.66-0.69)	0.60 (0.57-0.63)
	Discrimination slope	0.05	0.01
Calibration			
	Calibration intercept	-2.91	-2.53
	Calibration slope	0.09	0.05
	Hosmer-Lemeshow	χ2 = 0.47 (P=0.88)	χ2= 0.59 (P=0.87)

**Figure 2:**
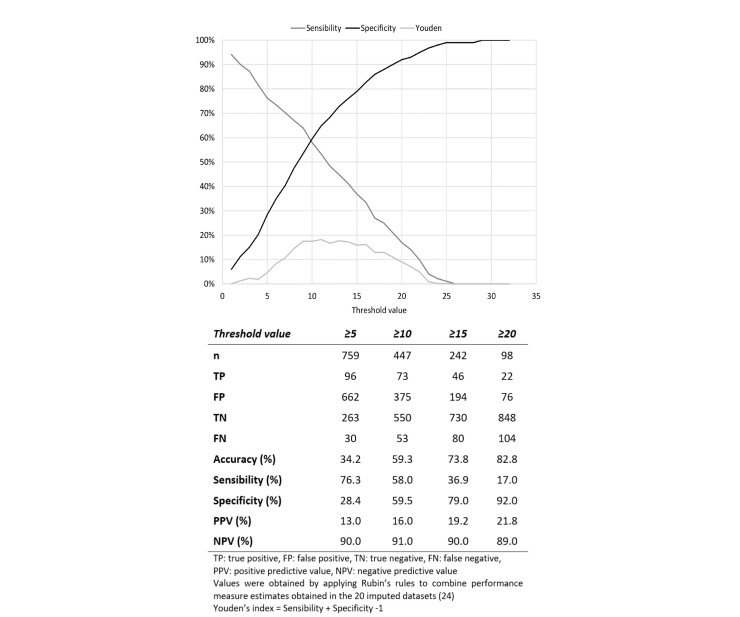
Classification performance of the risk score in the validation sample (N=1051). [TP=true positive; FP=false positive; TN=true negative; FN=false negative; PPV=positive predictive value; NPV=negative predictive value. Vales were obtained by applying Rubin's rules to combine performance measure estimates obtained in the 20 imputed datasets (24): Youden's index=sensitivity + specificity -1.]

In classification terms, the range of optimal threshold values was 10–15 (figure 2). A cut-off value of 10 for the score led in the validation sample to a diagnostic accuracy of 59%, a sensitivity of 58%, a specificity of 60%, a positive predictive value (PPV) of 16% but a negative predictive value (NPV) of 91%. Using a cut-off value of 15 provided greater diagnostic accuracy and similar NPV by excluding the presence of UEMSD in a higher proportion of workers, but also with a slight increase in false negatives.

### Prognostic value of the prediction model

Among the 1284 followed-up participants with complete data, the performance to predict incident UEMSD at follow-up clinical examination (prognostic performance) were quite similar in comparison to diagnostic performance, including the discriminant prognostic capacity: AUC 0.60, 95% CI 0.56–0.65 for the developed score (supplementary figure S1). The mean and median time from inclusion in the cohort to follow-up examination was 5.6 years.

### Sensitivity analyses

Similar predictive performances were obtained from complete case analysis in the validation sample (supplementary table S2). The performance in terms of classification in complete cases were also very close to those found on the imputed data (supplementary figure S2).

The prognostic ability of the developed score was further assessed separately for the three main locations of UEMSD. The discriminant performance of the score varied according to the location of MSD: for shoulder disorders (AUC 0.57, 95% CI 0.51–0.63), for elbow disorders (AUC 0.62, 95% CI 0.53–0.70) and for hand/wrist disorders (AUC 0.67, 95% CI 0.59–0.75).

## Discussion

### Summary of results

In this study, we developed and validated a scoring system to quantify the risk of clinically diagnosed UEMSD based on occupational factors. This predictive score including 6 physical exposures, 3 psychosocial work variables, and 4 factors related to work organization showed adequate calibration, poor discrimination but high negative predictive value in both the diagnostic and prognostic setting. These performance results, especially the predictive values, may however be related to the UEMSD prevalence in the studied sample.

### Work-related predictors in the risk score

The developed risk score includes a broad field of work-related factors (physical, psychosocial and organizational), which is consistent with a multifactorial origin of UEMSD (3, 25). According to systematic literature reviews (3, 26–29), there is reasonable evidence for causal relationship of physical exposures, such as heavy physical work, repetitive work and awkward postures, with at least one localization of UEMSD. Among the physical factors, biomechanical constraints at work may induce MSD through overuse of muscles and tendons (25, 30). The risk score for UEMSD obtained by multivariate modeling includes as predictors high perceived physical exertion, high repetitiveness of tasks, and repetitive/sustained awkward postures (arms above the shoulder, arms abduction, elbow flexion/extension and wrist twisting movements). The significant predictors in the present study are well-known risk factors for UEMSD (3, 26–29), including two risk factors for shoulder MSD, one risk factor for elbow MSD, one risk factor for wrist MSD, while repetitiveness and physical exertion are risk factors for the three localizations.

The inclusion of the lack of time to do work in the developed risk score is somewhat in agreement with the reported potential deleterious impact of high psychosocial work demands on UEMSD (3, 31). Our risk score also includes factors related to low social support by supervisor and co-workers. This is consistent with the literature on chronic musculoskeletal pain, despite the inconsistency of the epidemiological literature regarding the associations between occupational psychosocial exposures and UEMSD (14, 25, 31, 32). Van der Molen et al (27) concluded to low to very-low-quality evidence for an association between psychosocial job demands and the incidence of specific shoulder disorders. According to Dalboge et al (33), there is a lack of direct causal association between psychosocial exposure at work and subacromial impingement syndrome.

Work organization characteristics have effect on the biomechanical and psychosocial features of the working situations which workers encounter (25). Previous findings suggest indirect impacts of factors related to work organization on the risk of UEMSD (14, 15, 25, 31), it is a possible explanation of the lower weights assigned to organizational predictors in the scoring system. But, organizational factors are understudied in the epidemiological literature compared to the ergonomic literature (25, 34).

### Performance of the risk score

The developed UEMSD risk score showed comparable diagnostic and prognostic performances, with AUC ranging from 0.58 to 0.60 in validation samples. This relatively poor discrimination may be explained by the study sample characteristics and the low prevalence of UEMSD in this sample may also well explain the observed high negative predictive value. The score might perform differently in populations at higher risk of UEMSD. Indeed, a previous French study showed that an UEMSD risk score performed better in the construction sector which is characterized by a higher UEMSD prevalence than in general working population in which it was developed (35). Thus, it would be useful for further studies to investigate the performance of the developed score in working populations with a higher UEMSD prevalence. Moreover, a tool can be informative even with poor discriminatory performance according to reference standard values, ie, with AUC about 0.6 (36). The AUC measure does not account for misclassification costs resulting from false negative and false positive diagnoses. Additional studies using the net benefit approach are also required to determine the relevance of the score for stratifying workers according their risk of UEMSD, and more particularly to quantify whether the used of the developed score could lead to a net reduction in the number of investigation by an ergonomist among low-risk workers.

### Strengths and limitations

The methodology used in this study follows as much as possible the recommendations for the development and validation of diagnostic and prognostic prediction models (37, 38). The measurement of the outcome was based on a standardized diagnostic process defined by the European consensus SALTSA for six different types of UEMSD (13). Occupational factors were selected as candidate predictors based on clinical knowledge, literature reviews (3, 26–29, 31) and previous results on the French Cosali cohort (5, 11, 12, 14, 15). They were collected before the diagnostic clinical examination, and the exposures were subsequently defined according to recognized criteria (13). Missing data were handled by a multiple imputation process and a Lasso logistic regression model was applied to perform a parsimonious selection among all the candidate predictors tested in the prediction model. Using a penalized regression approach, a specific shrinkage factor could be applied to each predictor in the final model to reduce the potential model overfitting due to a ratio of events per variable <10, ie, 323 workers with UEMSD in the development sample and 47 candidate predictors tested. Another strength is the study population. On the one hand, the Cosali cohort included workers from several companies and covers many sectors of activity and occupations. It was considered as a good representation of the employed working population of the Pays de la Loire region (12). In addition, the size and design of the cohort made possible to constitute development and temporal validation samples, with similar characteristics. Finally, on a subsample, we were able to evaluate the prognostic values of the developed score, ie, their abilities to predict incident cases of UEMSD diagnosed at follow-up examination.

Several elements may explain the poor discriminatory capacity of our score. First, the low explained variance can be explained by the lack of important predictors in the final model. The developed risk score does not take into account known individual risk factors for UEMSD, such as obesity (5), since we aimed to develop a risk assessment tool related to occupational exposures only. We have previously shown that a significant proportion of UEMSD are attributable to occupational exposures, in particular to biomechanical and psychosocial factors (5). However, age and sex were forced into the Lasso logistic regression models in order to retain in the final model only occupational exposures that predict UEMSD independently of age and sex. This strategy has also been used in the development of a chronic low back pain risk score (39). Secondly, in order to obtain an easy-to-calculate risk score, we chose to base our prediction model on categorical exposure variables. Although we have used consensual exposure thresholds for each occupational factor, the dichotomization of these variables initially evaluated on a 4- or 5-point Likert scale has led to a loss of information which may have reduced their ability to predict UEMSD. Another concern is that the occupational exposures were collected by self-questionnaire and therefore self-assessed, which is a potential source of inaccuracy. In addition, these occupational exposure data were collected 20 years ago (2002–2003 and 2004–2005) and there may have been changes in UEMSD risk factors over time. Data from the national periodic Sumer survey show a contrasting evolution in the prevalence of occupational exposures among employees in France since the study period (40), thus the relative contribution of these factors in the UEMSD risk could have changed. Moreover, it is known that the risk of UEMSD increases with the accumulation over time/the chronicity of exposures, in particular for physical factors, while our score is based on exposures at the time of inclusion in the cohort and therefore does not take into account the duration nor history of exposures. Finally, the predicted outcome includes six different anatomical locations of UEMSD and the obtained prediction model may not be the best combination of predictors for each of the locations. However, this choice is in line with the ergonomic approach based on a global analysis of work situations (34, 41).

### Implications for clinical practice

The risk score developed in this study apprehends the upper limb in a global way without cutting by anatomical area (shoulder/elbow/wrist-hand) as proposed by some checklists (41), due to the existence of general risk factors for UEMSD common to the entire upper limb (repetitiveness, intensity of effort) and the need for a comprehensive approach to UEMSD risk assessment. This score focuses on the main work-related risk factors to be usable by non-medical interveners in the occupational setting and without requiring individual/personal medical information. Consequently, it does not take into account the individual risk factors for UEMSD. This kind of score might help to identify work situations that should benefit as a priority from a preventive intervention action at workplace according to the level of risk.

In other words, it could be used by prevention professionals as a first-line screening risk assessment tool to prioritize intervention: individuals / work situations classified as low risk of UEMSD by the score could benefit from additional investigations in second time, so that time-consuming and costly comprehensive risk management approach could target as a priority the more risky work situations. However, as mentioned earlier in the discussion, further external validation studies are needed to confirm the calibration and predictive performance of the developed score, as well as for determining its utility as a screening tool to stratify work situations according to their risk of UEMSD, especially among working populations with higher prevalence of UEMSD.

Such score could be also used to promote awareness of the risk of MSD by the companies, as highlighted in a previous qualitative study (35). Therefore, such an UEMSD risk score would be a relevant tool in the context of the implementation of the first stage of a hierarchical approach to work situations, such as Sobane method (42). However, the tool is not in itself a prevention approach, it is only one of the means of achieving it. Its use must be part of a prevention approach that involves all stakeholders (43), including particularly business managers.

### Concluding remarks

A work-related risk score for clinically diagnosed UEMSD was developed in this study, it includes physical, psychosocial and organizational work factors suggesting that comprehensive prevention approaches should be preferred to prevent UEMSD at the workplace. Such a score can be useful for raising awareness of the multifactorial risk of UEMSD at work and also for optimizing the costly and time-consuming interventional risk assessments by prevention professionals. Further studies are required to validate this score in other working populations, including in higher-risk subgroups, and to assess its practical usefulness.

### Ethics approval and consent to participate

Each worker provided informed written consent to participate in the Cosali study, and the study received the approval of the French Advisory Committee on the Processing of Information in Health Research (“CCTIRS”) and the National Committee for Data Protection (“CNIL”).

## Supplementary material

Supplementary materials
