# Supplementary material for: Development and validation of a work-related risk score for upper-extremity musculoskeletal disorders in a French working population
Source: Scand J Work Environ Health. 2023 Oct 31;49(8):558–68. doi: 10.5271/sjweh.4119 (PMC10858731; doi:10.5271/sjweh.4119)
Supplement: Supplementary materials [file SJWEH-49-558-S001.pdf]

Development and validation of a work-related risk score for upper-extremity musculoskeletal disorders in a French working population[1]

by Clémence Rapicault, MSc,[2] Yves Roquelaure, MD, PhD, Julie Bodin, PhD, Natacha Fouquet, PhD, Sandrine Bertrais, PhD

1. Supplementary materials
2. Correspondence to: Clémence Rapicault, INSERM UMR 1085 – IRSET, Equipe Epidémiologie en Santé au Travail et Ergonomie (ESTER), Faculté de Santé – Département Médecine, 28 rue Roger Amsler, CS 74521, F-49045 Angers Cedex 1, France. [E-mail: clemence.rapicault@univ-angers.fr]

Table of contents:

	page
Table S1: Comparison of occupational exposures between development and validation samples	2
Table S2: Performance of the risk score for identifying workers with UEMSD diagnosed at inclusion into the cohort. Results from complete case analysis in the validation sample (N=968)	7
Figure S1: Calibration plot showing the prognostic validity of the risk score in the subsample of workers without UEMSD at inclusion and with complete data. The distribution of predicted risks is shown at the bottom of the curve. The triangles indicate the observed frequencies by decile of the predicted risk.	8
Figure S2: Classification performance of the risk score for identifying workers with UEMSD diagnosed at inclusion into the cohort. Results from complete case analysis in the validation sample (N=968).	9

Table S1: Comparison of occupational exposures between development and validation samples

	Development sample (N=2468)	Validation sample (N=1051)	
	N (%)	N (%)	P-value[(a)]
**PHYSICAL FACTORS**			
**Repetitiveness of tasks (≥4h/day) with/without break**			0.071
missing	*15*	*14*	
No	1799 (73.3)	776 (74.8)	
Yes, without break	499 (20.4)	216 (20.8)	
Yes, with break	155 (6.3)	45 (4.4)	
**High perceived physical exertion (RPE Borg scale)**			0.780
missing	*9*	*7*	
No	1216 (49.5)	525 (50.3)	
Yes, 13≤RPE<15	715 (29.1)	291 (27.9)	
Yes, RPE≥15	528 (21.5)	227 (21.8)	
**Use of vibrating hand tools (≥2h/day)**			0.666
missing	*6*	*8*	
No	2142 (87)	913 (87.5)	
Yes	320 (13)	130 (12.5)	
**Exposure to cold temperature (≥4h/day)**			0.156
missing	*4*	*8*	
No	2303 (93.5)	988 (94.7)	
Yes	161 (6.5)	55 (5.3)	
**Use of computer keyboard or mouse (≥4h/day)**			0.541
missing	*3*	*9*	
No	1792 (72.7)	747 (71.7)	
Yes	673 (27.3)	295 (28.3)	
**Arms above shoulder level (≥2h/day)**			0.579
missing	*7*	*7*	
No	2128 (86.5)	910 (87.2)	
Yes	333 (13.5)	134 (12.8)	
**Holding hand behind the trunk (≥2h/day)**			0.318
missing	*6*	*9*	
No	2331 (94.7)	995 (95.5)	
Yes	131 (5.3)	47 (4.5)	
**Arms abduction (60–90°) (≥2h/day)**			0.606
missing	*5*	*7*	
No	2086 (84.7)	877 (84)	
Yes	377 (15.3)	167 (16)	
**Elbow flexion/extension movements (≥2h/day)**			0.496
missing	*8*	*9*	
No	1640 (66.7)	707 (67.8)	
Yes	820 (33.3)	335 (32.2)	
**Pronation and supination movements (≥2h/day)**			0.460

missing	*10*	*8*	
No	2095 (85.2)	899 (86.2)	
Yes	363 (14.8)	144 (13.8)	
**Wrist twisting movements (≥2h/day)**			0.005
missing	*22*	*22*	
No	1585 (64.8)	717 (69.7)	
Yes	861 (35.2)	312 (30.3)	
**Use of the pinch grip (≥4h/day)**			0.262
missing	*10*	*6*	
No	2254 (91.7)	970 (92.8)	
Yes	204 (8.3)	75 (7.2)	
**PSYCHOSOCIAL FACTORS: DECISION LATITUDE**			
**Learn new things**			0.698
missing	1	3	
Strongly disagree/disagree	276 (11.2)	122 (11.6)	
Agree/strongly agree	2191 (88.8)	926 (88.4)	
**Repetitive work**			0.049
missing	3	6	
Strongly disagree/disagree	606 (24.6)	290 (27.8)	
Agree/strongly agree	1859 (75.4)	755 (72.2)	
**Requires creativity**			0.286
missing	5	3	
Strongly disagree/disagree	891 (36.2)	399 (38.1)	
Agree/strongly agree	1572 (63.8)	649 (61.9)	
**Allows own decisions**			0.616
missing	6	3	
Strongly disagree/disagree	445 (18.1)	182 (17.4)	
Agree/strongly agree	2017 (81.9)	866 (82.6)	
**High skill level**			0.592
missing	8	9	
Strongly disagree/disagree	855 (34.8)	372 (35.7)	
Agree/strongly agree	1605 (65.2)	670 (64.3)	
**Little decision freedom**			0.077
missing	7	4	
Strongly disagree/disagree	1820 (74.0)	804 (76.8)	
Agree/strongly agree	641 (26.0)	243 (23.2)	
**Variety**			0.772
missing	5	2	
Strongly disagree/disagree	435 (17.7)	181 (17.3)	
Agree/strongly agree	2028 (82.3)	868 (82.7)	
**A lot to say**			0.597
missing	10	2	
Strongly disagree/disagree	623 (25.4)	257 (24.5)	
Agree/strongly agree	1835 (74.6)	792 (75.5)	
**Develop own abilities**			0.476
missing	8	4	

Strongly disagree/disagree	546 (22.2)	221 (21.1)	
Agree/strongly agree	1914 (77.8)	826 (78.9)	
**PSYCHOSOCIAL FACTORS: PSYCHOLOGICAL DEMANDS**			
**Work fast**			0.271
missing	*1*	*2*	
Strongly disagree/disagree	974 (39.5)	435 (41.5)	
Agree/strongly agree	1493 (60.5)	614 (58.5)	
**Work hard**			0.045
missing	*9*	*2*	
Strongly disagree/disagree	922 (37.5)	431 (41.1)	
Agree/strongly agree	1537 (62.5)	618 (58.9)	
**No excessive work**			0.590
missing	*7*	*2*	
Strongly disagree/disagree	1414 (57.5)	613 (58.4)	
Agree/strongly agree	1047 (42.5)	436 (41.6)	
**Enough time**			0.994
missing	*3*	*4*	
Strongly disagree/disagree	633 (25.7)	269 (25.7)	
Agree/strongly agree	1832 (74.3)	778 (74.3)	
**Conflicting demands**			0.039
missing	*8*	*4*	
Strongly disagree/disagree	1739 (70.7)	776 (74.1)	
Agree/strongly agree	721 (29.3)	271 (25.9)	
**Intense concentration**			0.807
missing	*10*	*3*	
Strongly disagree/disagree	1287 (52.4)	544 (51.9)	
Agree/strongly agree	1171 (47.6)	504 (48.1)	
**Tasks interrupted**			0.390
missing	*6*	*2*	
Strongly disagree/disagree	1151 (46.7)	507 (48.3)	
Agree/strongly agree	1311 (53.3)	542 (51.7)	
**Hectic work**			0.904
missing	*13*	*1*	
Strongly disagree/disagree	1477 (60.2)	634 (60.4)	
Agree/strongly agree	978 (39.8)	416 (39.6)	
**Wait on others**			0.262
missing	*9*	*3*	
Strongly disagree/disagree	1805 (73.4)	750 (71.6)	
Agree/strongly agree	654 (26.6)	298 (28.4)	
**PSYCHOSOCIAL FACTORS : SOCIAL SUPPORT**			
**Supervisor concerned**			0.440
missing	*25*	*18*	
Strongly disagree/disagree	660 (27)	266 (25.7)	
Agree/strongly agree	1783 (73)	767 (74.3)	
**Supervisor pays attention**			0.602
missing	*17*	*14*	

Strongly disagree/disagree	458 (18.7)	186 (17.9)	
Agree/strongly agree	1993 (81.3)	851 (82.1)	
**Helpful supervisor**			0.871
missing	*17*	*13*	
Strongly disagree/disagree	537 (21.9)	230 (22.2)	
Agree/strongly agree	1914 (78.1)	808 (77.8)	
**Supervisor good organizer**			0.370
missing	*33*	*21*	
Strongly disagree/disagree	492 (20.2)	222 (21.5)	
Agree/strongly agree	1943 (79.8)	808 (78.5)	
**Co-workers competent**			0.190
missing	*25*	*18*	
Strongly disagree/disagree	181 (7.4)	90 (8.7)	
Agree/strongly agree	2262 (92.6)	943 (91.3)	
**Co-workers interested in me**			0.392
missing	*33*	*20*	
Strongly disagree/disagree	277 (11.4)	107 (10.4)	
Agree/strongly agree	2158 (88.6)	924 (89.6)	
**Friendly co-workers**			0.802
missing	*28*	*18*	
Strongly disagree/disagree	169 (6.9)	74 (7.2)	
Agree/strongly agree	2271 (93.1)	959 (92.8)	
**Co-workers helpful**			0.532
missing	*27*	*17*	
Strongly disagree/disagree	241 (9.9)	95 (9.2)	
Agree/strongly agree	2200 (90.1)	939 (90.8)	
**ORGANIZATIONAL FACTORS**			
**Temporary employment**			0.093
missing	*8*	*5*	
No	2213 (90)	960 (91.8)	
Yes	247 (10)	86 (8.2)	
**Shift work**			0.163
missing	*13*	*11*	
No	2063 (84)	854 (82.1)	
Yes	392 (16)	186 (17.9)	
**Irregular working hours**			0.022
missing	*14*	*6*	
No	1632 (66.5)	653 (62.5)	
Yes	822 (33.5)	392 (37.5)	
**Work with temporary workers**			0.328
missing	*6*	*5*	
No	1720 (69.9)	748 (71.5)	
Yes	742 (30.1)	298 (28.5)	
**Work pace dependent on automatic rate**			0.877
missing	*85*	*8*	
No	2011 (84.4)	878 (84.2)	

Yes	372 (15.6)	165 (15.8)	
**Work pace dependent on colleague's work**			0.162
missing	*102*	*10*	
No	1659 (70.1)	705 (67.7)	
Yes	707 (29.9)	336 (32.3)	
**Work pace dependent on production standards or deadlines**			0.121
missing	*84*	*9*	
No	1256 (52.7)	519 (49.8)	
Yes	1128 (47.3)	523 (50.2)	
**Work pace dependent on external demand**			<0.001
missing	*56*	*8*	
No	1419 (58.8)	489 (46.9)	
Yes	993 (41.2)	554 (53.1)	
**Work pace imposed by permanent monitoring**			0.098
missing	*92*	*10*	
No	1739 (73.2)	790 (75.9)	
Yes	637 (26.8)	251 (24.1)	

RPE Borg scale: Rating perceived exertion Borg scale
[a] Chi-square test

Table S2: Performance of the risk score for identifying workers with UEMSD diagnosed at inclusion into the cohort. Results from complete case analysis in the validation sample (N=968).

Global performance	
R^2 (Nagelkerke)	2.1 %
Brier score	0.10
Brier score (scaled)	1.1 %
Discrimination	
AUC	0.59 (0.53-0.65)
Discrimination slope	0.01
Calibration	
Calibration intercept	-2.50
Calibration slope	0.05
Hosmer-Lemeshow	χ^2 = 6.53, p = 0.59

Figure S1: Calibration plot showing the prognostic validity of the risk score in the subsample of workers without UEMSD at inclusion and with complete data. The distribution of predicted risks is shown at the bottom of the curve. The triangles indicate the observed frequencies by decile of the predicted risk.

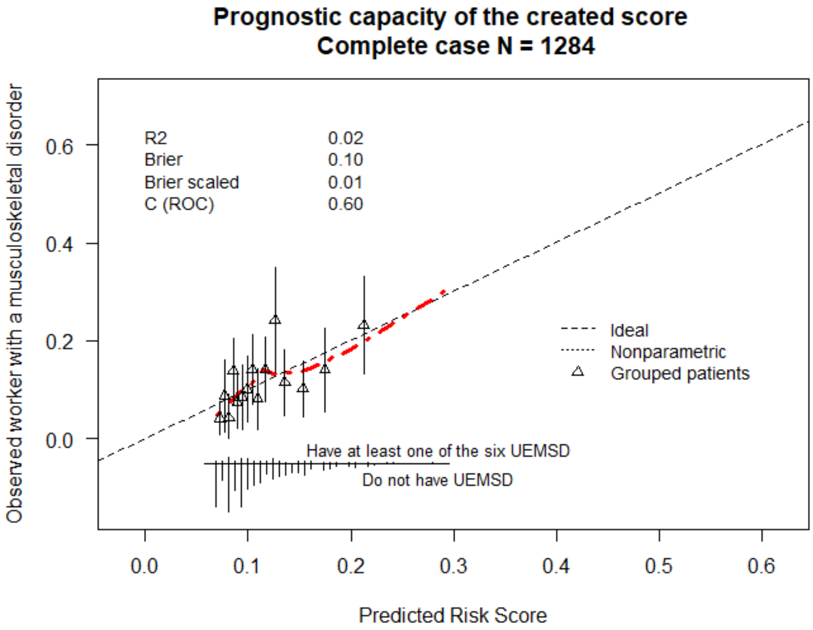

Figure S2: Classification performance of the risk score for identifying workers with UEMSD diagnosed at inclusion into the cohort. Results from complete case analysis in the validation sample (N=968).

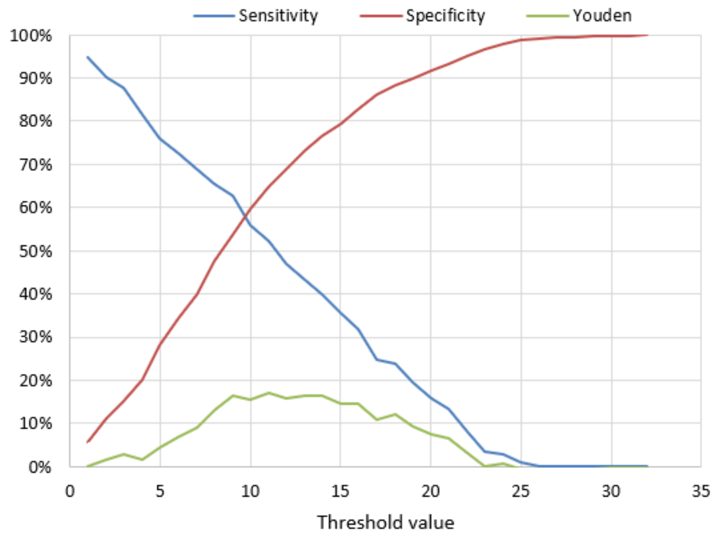

Threshold value	*≥5*	*≥10*	*≥15*	*≥20*
**n**	700	408	217	89
**TP**	86	63	40	18
**FP**	614	345	177	71
**TN**	241	510	678	784
**FN**	27	50	73	95
**Accuracy (%)**	33.8	59.2	74.2	82.9
**Sensitivity (%)**	76.1	55.8	35.4	15.9
**Specificity (%)**	28.2	59.6	79.3	91.7
**PPV (%)**	12.3	15.4	18.4	20.2
**NPV (%)**	89.9	91.1	90.3	89.2

TP: true positive, FP: false positive, TN: true negative, FN: false negative, PPV: positive predictive value, NPV: negative predictive value

Youden's index = Sensitivity + Specificity -1
